# Has open data arrived at the *British Medical Journal (BMJ)*? An observational study

**DOI:** 10.1136/bmjopen-2016-011784

**Published:** 2016-10-13

**Authors:** Anisa Rowhani-Farid, Adrian G Barnett

**Affiliations:** Australian Centre for Health Services Innovation (AusHSI), Institute of Health and Biomedical Innovation (IHBI), Queensland University of Technology (QUT), Brisbane, Queensland, Australia

**Keywords:** Data Sharing, Open Data, Open Science, Research Integrity, Reproducibility

## Abstract

**Objective:**

To quantify data sharing trends and data sharing policy compliance at the *British Medical Journal (BMJ)* by analysing the rate of data sharing practices, and investigate attitudes and examine barriers towards data sharing.

**Design:**

Observational study.

**Setting:**

The *BMJ* research archive.

**Participants:**

160 randomly sampled *BMJ* research articles from 2009 to 2015, excluding meta-analysis and systematic reviews.

**Main outcome measures:**

Percentages of research articles that indicated the availability of their raw data sets in their data sharing statements, and those that easily made their data sets available on request.

**Results:**

3 articles contained the data in the article. 50 out of 157 (32%) remaining articles indicated the availability of their data sets. 12 used publicly available data and the remaining 38 were sent email requests to access their data sets. Only 1 publicly available data set could be accessed and only 6 out of 38 shared their data via email. So only 7/157 research articles shared their data sets, 4.5% (95% CI 1.8% to 9%). For 21 clinical trials bound by the *BMJ* data sharing policy, the per cent shared was 24% (8% to 47%).

**Conclusions:**

Despite the *BMJ*'s strong data sharing policy, sharing rates are low. Possible explanations for low data sharing rates could be: the wording of the *BMJ* data sharing policy, which leaves room for individual interpretation and possible loopholes; that our email requests ended up in researchers spam folders; and that researchers are not rewarded for sharing their data. It might be time for a more effective data sharing policy and better incentives for health and medical researchers to share their data.

**Strengths and limitations of this study**

- Our study quantified data sharing among all types of research articles published in the *British Medical Journal (BMJ)* from 2009 to 2015.
- The *BMJ* data sharing policy specifically applies to clinical trial data, but our study analysed data sharing among all studies that have original raw data.
- The sample size was 160 articles, which is relatively small.
- The *BMJ* data sharing policy suggests using the *BMJ* as a broker to negotiate data access; however, we did not use this service given the amount of time and resources it required both on our part and on the *BMJ*'s.

## Introduction

Open data are defined as ‘available, intelligible, assessable and useable data’.^1^ The practice of open data or ‘data sharing’ is the term given to the exercise of making all raw data fully and openly available, creating transparency and ensuring reproducibility, and driving further discovery by allowing new knowledge to be generated in the context of earlier discoveries.^2–4^ Though the concept of data sharing has only recently been identified as a subtheme of metaresearch,^5^ it was a research topic 15 years ago when Reidpath and Allotey conducted a prospective study to examine data sharing among *BMJ* articles. Only 1 out of 29 researchers contacted (3%) made their data sets available. The reluctance of researchers to make their data available raised questions about the validity of their findings, and suggested the researchers were potentially more concerned with not losing an advantage than advancing science through data sharing.^6^

The research climate in 2001 was significantly different to the current era, where rapid technological advances are contributing to what Bartling and Friesike^7^ refer to as the second scientific revolution with terms such as ‘data sharing’, ‘open data’, ‘open research’ and ‘Science 2.0’ proliferating in the scientific discourse. Open data and data sharing are now being considered as fundamental elements of the shift towards research that is verifiable, reproducible and transparent.^8^

Given the many recent changes to research publishing, it is fitting to conduct a similar study to Reidpath and Allotey's. Our study quantified data sharing trends at the *BMJ* from 2009 to 2015, paying particular attention to policy changes at the journal that aimed to increase data sharing.

We selected the *BMJ* because it is an international health and medical research journal leading the data sharing movement. In March 2009, the *BMJ* introduced the idea of a data sharing statement in research articles. The purpose was to explain whether there were any additional data available and how they could be accessed.^9^ The *BMJ* was among one of the first medical journals to introduce such a concept; a significant milestone in the data sharing movement that is gathering momentum in health and medical research. In 2010, the *BMJ* crystallised the data sharing statement into a policy.^10^ In 2012, the *BMJ* introduced a stricter data sharing policy for drugs and device trials: ‘from 1 January 2013, trials of drugs and medical devices will be considered for publication only if the authors commit to making the relevant anonymised patient-level data available on reasonable request’.^11^ From 1 July 2015 the *BMJ*'s requirements for data sharing extended to all submitted clinical trials, not just those that tested drugs or devices.^12^ A number of journals now require authors share their data,^13^ ^14^ either via a public repository or making it freely available on request. The success of these policies remain largely untested.^15^

Although the *BMJ*'s data sharing policy focuses on trials, data sharing should ideally apply to all types of research. This idea forms the basis of our study, which not only examined clinical trials, but included all types of studies with original raw data. The reason behind our approach is that the *BMJ* is “…keen to maximise the usefulness and usage of data and promote transparency, and to satisfy the requirements of the many research funders that encourage or even mandate data sharing”.^16^ From this statement, we deduce that the *BMJ* supports research reproducibility and transparency of research findings, which support high-quality research and apply to all research data.

## Methods

### Overview

A random sample of research papers published in the *BMJ* were examined to observe the issues arising with data sharing, including the point that was raised on a recent *BMJ* podcast,^17^ namely, that researchers indicate the availability of their data in order to pass the editorial review, but fail to share when it is requested. We contacted researchers who indicated in their data sharing statements that they were willing to make their data sets available. Our aims were to: (1) estimate the rate of data sharing, and (2) examine the shared data sets by comparing them to the published findings to quantify the integrity of the data sharing process.

### Participants

A random number generator was used to select the research papers (using Excel). We excluded studies whose complete data were available in the article, including systematic reviews and meta-analyses. All other types of studies were included. Twenty *BMJ* research papers were randomly sampled per year from 2009 to 2014. In 2015, we randomly selected 20 papers before a major policy change on 1 July 2015 and 20 papers after. The total sample size was 160. We did not use a formal sample size calculation because they are often limited.^18^ Instead, the sample size was based on the practical considerations of reading papers, contacting authors and examining their data.

The setting of this study was the *BMJ* research archive. All information required for data collection was publicly available online. Data collection was started on 12 November 2015 and ended on 31 January 2016. The first author (AR-F) read the research papers and extracted the details of authors. The following variables were documented: type of study, data sharing statement and data availability. The second author (AGB) independently assessed the data sharing statements for 20 randomly selected articles. No disagreements were found, meaning that there is a 90% probability that the agreement between the two authors is over 90%.

Authors of articles who stated a willingness to share their data were contacted via email. A de-identified copy of our approach email to authors is included as an online supplementary web appendix. Three research articles had their data within the text of the article itself, these researchers were not contacted, reducing the sample size from 160 to 157.

10.1136/bmjopen-2016-011784.supp1 supplementary web appendix

Email requests for data were sent from 18 November 2015 to 16 December 2015. The 28 January 2016 was set as the final date for receiving data sets. A single reminder was sent to researchers who made an initial positive response but who did not send their data sets even after 2 weeks. Alternative email addresses were only sought when our original email bounced. A response from authors was taken as consent to participate in the study—all authors were informed about the ethical approval of the study and the procedure of consent.

Some research articles indicated that their data were available from external sources but were subject to additional applications. We did not apply for these data sets given the large amount of time it would take to apply, and because there was no guarantee we would gain access to the data.

### Quantitative variables

We first categorised each article into:

- *Data not available*—research articles whose data sharing statement was that ‘no additional data are available’;
- *Data available*—research articles that indicated in their data sharing statement that their data are available.

And then categorised those with data available into:

- *Data not available*—research articles that did not make their data sets available to our team on request and research articles that had ‘publicly available data’ that we could not locate;
- *Data potentially available*—research articles that indicated that their data sets were available but they were subject to forms and applications and research articles that mentioned that their data sets were publicly available but they were not easily accessible and which also required forms and applications;
- *Data easily available (received)*—the research articles that made their data set available to our team.

### Statistical methods

We reported the per cent of data sharing and 95% CI. We examined the sample sizes and variables in the received data to verify that they matched the original paper. We used logistic regression to examine a change in data sharing over time using publication date as the time variable. We used a log link in place of the logit, so our results are prevalence ratios not ORs.^19^

## Results

### Participants

Out of the 157 randomly sampled research articles, 50 indicated in various ways the availability of their raw data. The numbers grouped by what was written in the data sharing statements are given in table 1.

**Table 1:** Numbers of various data sharing statements for randomly selected *British Medical Journal (BMJ)* research articles (2009–2015) that indicated the availability of their raw data

	2009	2010	2011	2012	2013	2014	2015 (1)	2015 (2)	Total
Additional data available from author	1	1	3	4	3	2	5	0	19
Reasonable requests for access to data can be made to the authors	0	1	0	2	1	2	7	2	15
Data were available from external sources subject to additional applications	0	1	0	1	0	1	3	2	8
Data publicly available	0	0	0	0	0	2	0	3	5
Data were available once they had completed all planned analyses and published results	0	1	0	1	0	0	0	0	2
Data were available after 3 years, subject to a contract and authors will examine requests	0	0	0	0	0	1	0	0	1
Total	1	4	3	8	4	8	15	7	50

Thirty-eight emails were sent to researchers who indicated in some way that their data sets were available. Of the 38 authors who were emailed, only 16 of them responded to our email, leaving 22 non-responses, which were categorised as ‘data not available’. Six of the 16 responses provided their data sets to our team (1 of which was a randomised clinical trial (RCT) but we could not verify that the data shared matched the article), these articles were categorised as ‘data easily available (received)’. Eight of the 16 responses raised caveats on request, 3 of which were categorised as ‘data potentially available’ as they were subject to forms and applications, and the remaining 5 were categorised as ‘data not available’. Two responses never followed through to make their data available and were categorised as ‘data not available’.

Twelve research articles had data that were available publicly or subject to forms: three provided external links that were no longer functioning, and three provided generalised links with no clear indication of the specific data set that was used for the purpose of the study. These six articles were categorised as ‘data not available’. Five of the 12 articles were subject to application forms; these articles were categorised as ‘data potentially available’. Only one of the ‘publicly available’ data sets was uploaded onto a public data depository, Dryad.

Out of the 50 articles that had data available, 21 were RCTs. One RCT data set was freely available on Dryad, leaving 20 RCTs which were emailed to request their data. Thirteen of the 20 did not respond to our email and were categorised as ‘data not available’, 4/20 made their data available (1 of which was unverifiable) an overall sharing rate of 24% (8% to 47%). The remaining 3/7 responses raised caveats and did not make their data available to our team.

**Figure 1:**
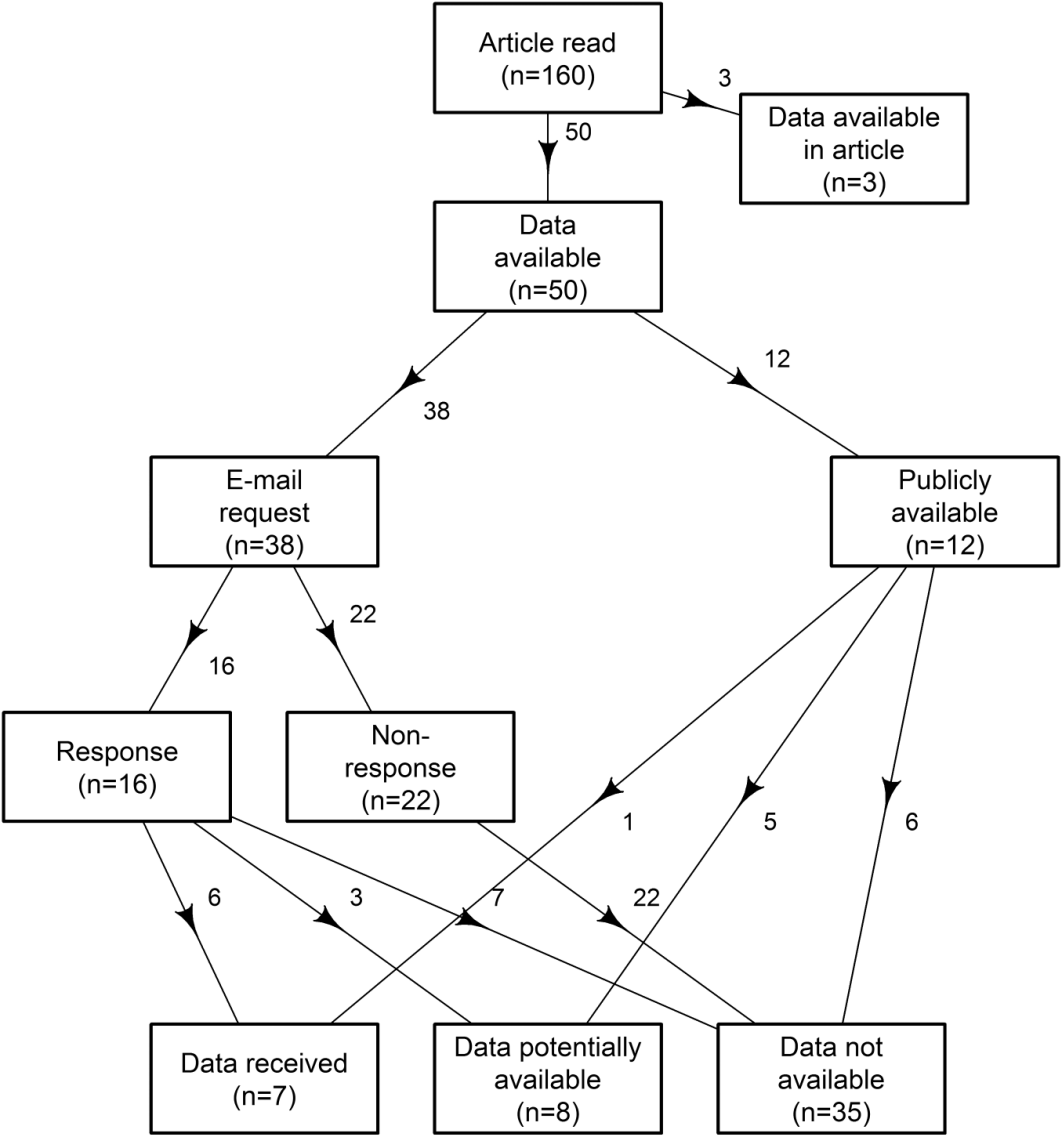
Flow chart of the randomly sampled *BMJ* research articles showing the availability of data. *BMJ, British Medical Journal*.

**Figure 2:**
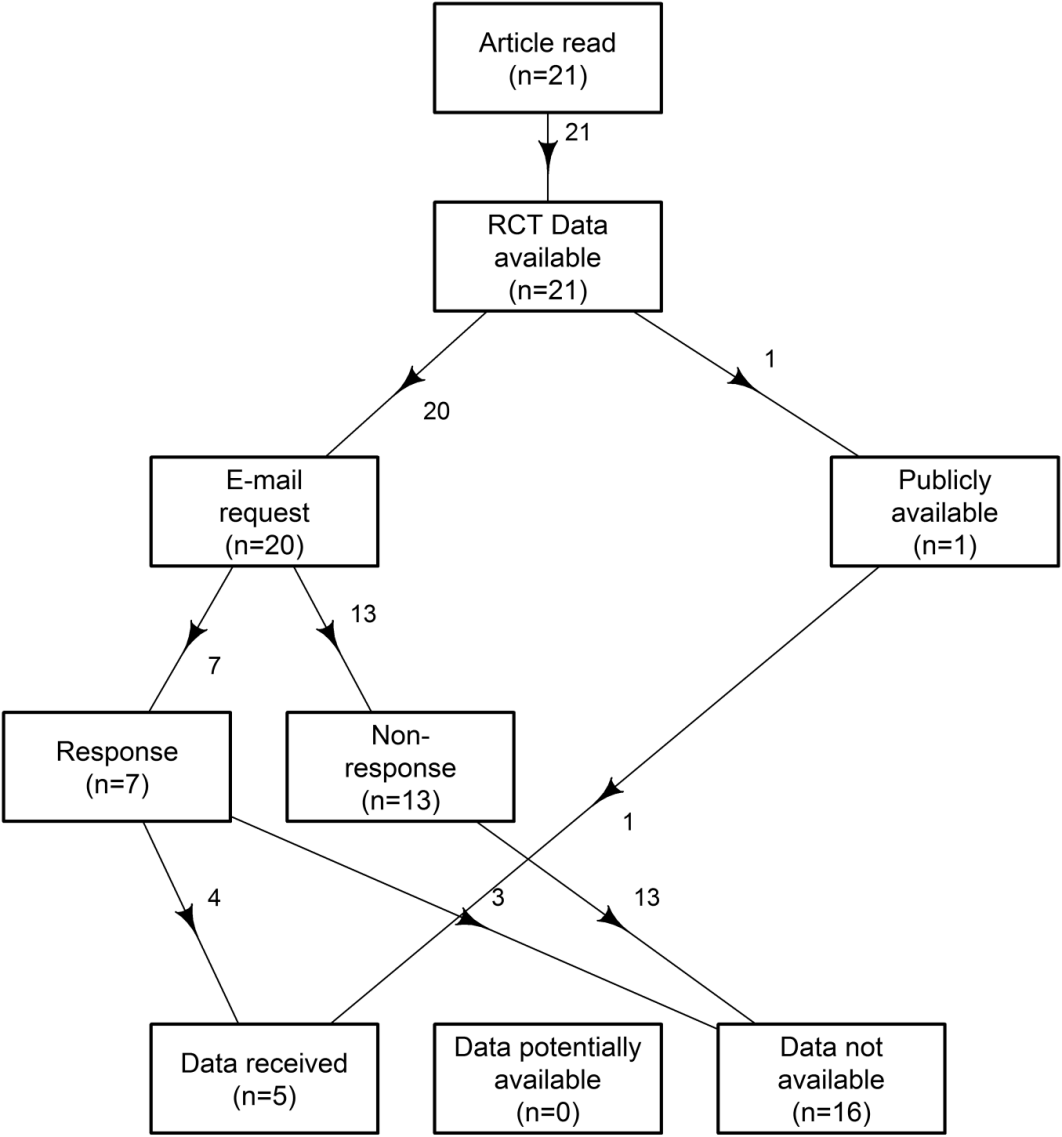
Flow chart of the randomly sampled *BMJ* research articles bound by the *BMJ* data sharing policy, RCTs, showing the availability of data. *BMJ, British Medical Journal*; RCT, randomised clinical trial.

**Figure 3:**
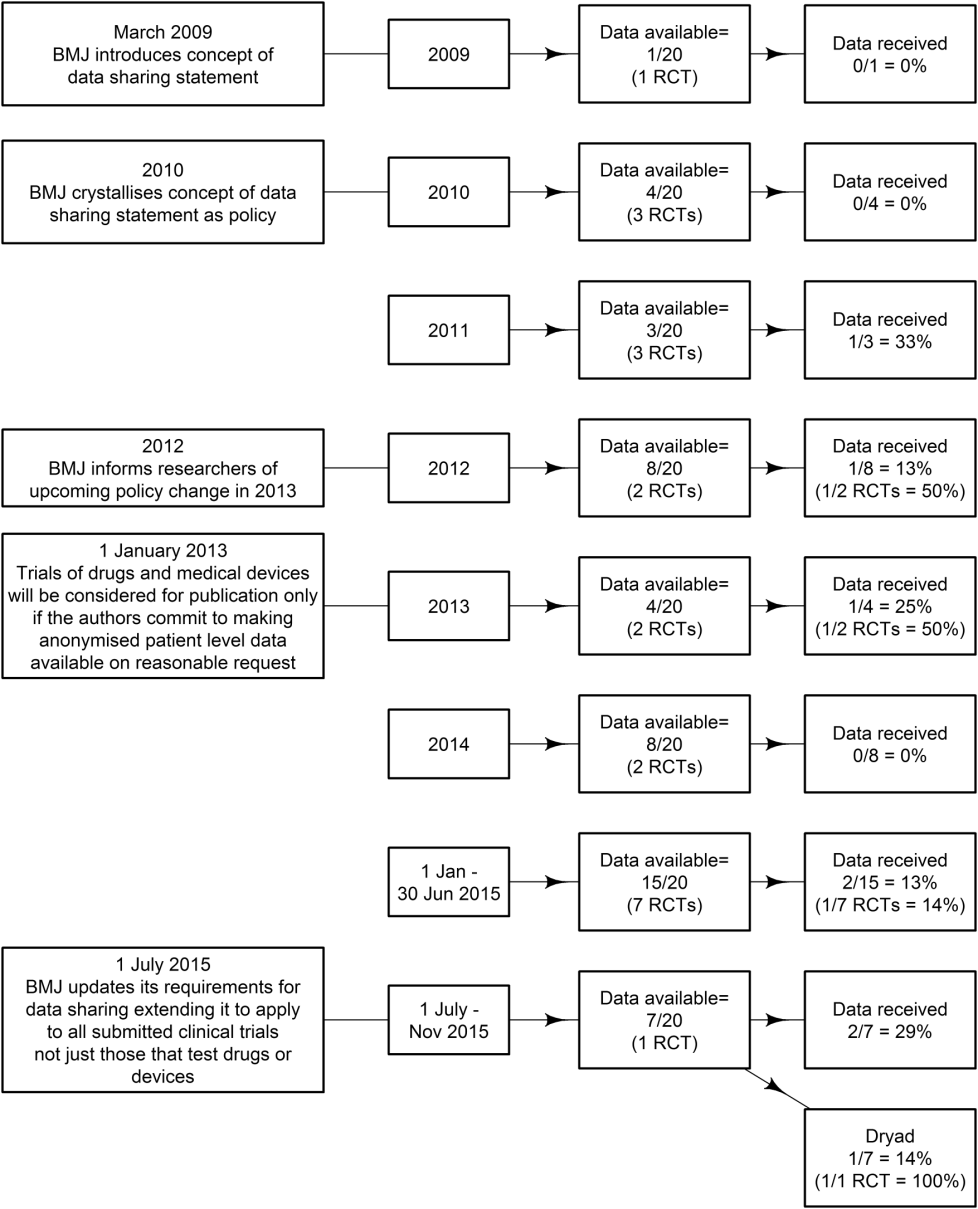
Summary of data availability and actual data received for BMJ research articles grouped by year and in relation to data sharing policy changes. *BMJ, British Medical Journal*; RCT, randomised clinical trial.

A flow chart of the data sharing results is shown in figures 1 and 2 for RCTs which are bound by the *BMJ* data sharing policy. The data sharing rates by *BMJ* policy changes are in figure 3.

### Main results

The total numbers were: 7/50 articles had ‘data easily available (received)’, 35/50 articles were ‘data not available’ and 8/50 articles were ‘data potentially available’.

Six of the seven data sets contained data that matched the article, with one data set unverifiable as it was difficult to navigate the data and no data dictionary was provided.

The percentage of data easily available from the 157 articles was only 4.5% (95% CI 1.8% to 9%). One of the shared data sets was not verifiable, so the actual data sharing rate might be lower than 4.5%. A further eight articles had data potentially available, so the data sharing rate could be as high as 9.6% (5.5% to 15%).

For RCTs, 5/21 RCTs made their data sets easily available, a data sharing rate of 24% (95% CI 8% to 47%). Sixteen of the 21 RCTs were categorised as ‘data not available’, and 0/21 in ‘data potentially available’.

Twenty-nine of the 50 articles were not bound by the policy but indicated data availability in their data sharing statements, only 2 of which made their data available. The sharing rate for those articles not bound by the *BMJ* data sharing policy is: 2/29, 7% (95% CI 1% to 23%).

### Authors’ responses to data sharing

The authors who made their data sets available did so with positive and encouraging words. Here are a few examples:

Good luck with your project, I am a firm supporter of open access to data.

Thank you very much for you interest in our study. We adhere the BMJ data sharing policy indeed. Please find attached the data files.

One researcher went so far as to offer to translate the data set into English.

Eight out of 16 authors provided email responses that were not consistent with their data sharing statements and raised caveats, including the requirement for entering into contracts with their institutions; writing a detailed plan indicating what we will do with their data; potentially paying for their data; that their data were no longer available as they are carrying out additional studies and that their data were only available on their own university premises. These hidden policies, contracts, costs and rules were not included in their *BMJ* data sharing statements. One researcher thought our research question was not ‘a reasonable research question’ and so refused to share their data.

### Change over time

A logistic regression showed that there was a 26% increase in the rate of ‘data shared’ for every additional year between 2009 and 2015 (95% CI 13% to 43%), and a 40% increase in the rate of ‘data promised’ for every additional year between 2009 and 2015 (95% CI −4% to 131%).

## Discussion

Only 32% of research articles published indicated the availability of their raw data. And then only 14% of those approached made their data easily available, and just one was freely accessible on Dryad. This gives an overall per cent of only 4.5% of data sharing for research articles at the *BMJ*, with a higher 24% data sharing rate among clinical trials that are bound by the *BMJ* data sharing policy.

### Interpretation

From the 50 out of 157 authors that indicated the availability of their raw data, less than half were clinical trials (21), and the rest were: cohort studies, cross-sectional analyses, modelling studies, case–control studies, retrospective analyses and others. It is encouraging to note that the majority of research articles that offered to make their raw data available were not bound by the data sharing policy that specifically applies to clinical trials. Assessing compliance of the *BMJ* data sharing policy was not the focus of our study as we were interested in all types of research articles. The easily available data sharing rate for clinical trials was 24%, which is higher than the rate for all articles types, but still low.

There are of course cases where ethical and legal constraints prevent data sharing, and we did not measure these occurrences.

Though 50 out of 157 research articles indicated the availability of their raw data, only 7 researchers easily provided their data for this study. It seems data sharing rates at the *BMJ* have only increased from 3% to 4.5% in 15 years, but with a 40% increase in the rate of ‘data promised’ since 2009^6^ demonstrating an increased compliance with data sharing policies for publication purposes, but not in practice.^17^

With regard to the caveats that were raised only after we requested access to ‘available’ raw data, we recognise that researchers have the right to set their own conditions for data access, but none of these conditions were mentioned in the data sharing statements. Ideally authors should state all the conditions in their data sharing statement, so as to clearly outline the procedures for accessing their raw data. It should not take much extra time to add this information to the data sharing statement. If there are restrictions on data availability—such as, home institution restrictions or other agreements with companies—these restrictions should be clearly outlined in the data sharing statement, an example of which could read: ‘our university's data sharing policy is that data are only available at our institution’. Ideal data sharing is freely available, easily accessible raw data that are downloadable from an online data depository, such as Dryad.

Our findings are comparable to similar studies assessing data sharing rates at *Public Library of Science (PLoS)* journals. A study by Savage and Vickers^20^ in 2009 received only 1/10 data sets (10%) that were requested, and a larger sample of 441 biomedical journal articles published from 2000 to 2014 had a data sharing rate of 0%, although these researchers only searched for freely available data and did not email authors.^5^ It is evident that data sharing is not common practice even among publishers with strong data sharing policies such as the *BMJ* and *PLoS*.

The cultural shift towards more open data in health and medical research is not as developed as the discipline of genomics. An empirical study conducted by Milia *et al*^21^ in 2012 demonstrated that ‘the majority of published data regarding human genetic variation are made openly available to the scientific community’.

There are a few possible explanations for the low data sharing rates at the *BMJ*. The wording of the *BMJ* data sharing policy states that authors of all submitted clinical trials, not just those that test drugs and devices, commit to making the relevant anonymised patient-level data available on reasonable request.^12^ Fiona Godlee's editorial post in 2012 explains ‘reasonable request’ as:

“As for ‘reasonable request’, The BMJ is not in a position to adjudicate, but we will expect requesters to submit a protocol for their re-analysis to the authors and to commit to making their results public. We will encourage those requesting data to send a rapid response to *thebmj.com*, describing what they are looking for. If the request is refused we will ask the authors of the paper to explain why.”^11^

The interpretation of ‘reasonable request’ is left to individual authors. What we thought as a ‘reasonable request’ may not be by other researchers, and could be behind the low data sharing rate. Some thought that the purpose of our study was not worth their time and resources, hence labelling our study in the category of unreasonable requests. It is not the purpose of this paper to convince the audience of the reasonability of our study, rather to bring to the *BMJ*'s attention the ambiguity created by the policy wording. With regard to submitting a protocol, our email included all the procedures of our examination of the data set for verification. We did not use the *BMJ* to broker access to papers on our behalf, and the data sharing rate could be higher if we used this route, although we note that this takes additional time and effort on our behalf and staff at the *BMJ*.

There are other potential reasons for the low data sharing rates. Given that 55% of the researchers contacted via email did not respond, we could deduce that: they never received our email due to out-dated email addresses or spam filters, that researchers were too busy, or that our request was simply ignored. We therefore recommend that multiple contacts are given, potentially including other researchers or even Twitter accounts. Non-response problems would be overcome by having the data stored by a third party, such as Dryad, as recommended by the *BMJ*.

Another possible barrier of data sharing is the lack of rewards in the scientific community. Researchers who participate in the culture of sharing should be supported and rewarded by the academic and research career systems.^22–24^ The lack of incentives for data sharing is a key barrier as researchers are often time poor and many do not see the value of spending time preparing their data or may be concerned about lengthy follow-up questions. A recent study conducted by Kidwell *et al*^25^ demonstrated that badges, developed by the Centre of Open Science, were effective incentives that increased data sharing rates. To encourage data sharing in health and medical research, it might be beneficial to change the criteria by which scientists and their teams are rewarded for their efforts by funding agencies and institutions.^26^ Ioannidis and Khoury^26^ designed the ‘PQRST approach’ for rewarding researchers, where the ‘S’ stood for sharing of data, code and protocols. To contribute to the adoption of a culture of data sharing, in early 2016 the International Committee of Medical Journal Editors (ICMJE) put together a proposal outlining some requirements to help meet the mandating of clinical trial data sharing worldwide.^27^

### Limitations

The sample size of 160 is relatively small. However, the CI for the rate of easily shared data are quite narrow and the upper limit is below 10%.

Our data sharing rate could be increased by more active chasing of researchers, yet, as Iqbal *et al*^5^ indicated, ‘the yield would be uncertain, and personal communications should not replace the lack of transparency in the published scientific record’. As such, we did not try to find alternative email addresses for those researchers who did not respond (we did try to find an alternative address if an email bounced), nor did we follow-up on them. Also, we did not approach the journal to help us negotiate access due to the amount of time and resources such a task requires both on our part and the *BMJ*'s for up to 160 papers.

We did not compare the characteristics of those who did and did not share their data (eg, which country was best/worst) as that was not one of our study aims.

### Generalisability

We used a random sample of *BMJ* research papers and only excluded meta-analyses and systematic reviews. Hence, our results should be generalisable to the wider *BMJ* literature and potentially to other general medical journals.

## Conclusion

As policies and procedures, rules and regulations that promote and encourage data sharing become more common, our study provides a glimpse into the reality of data sharing practices among health and medical researchers, using the *BMJ* as a case study. Has open data arrived at the *BMJ*? We think not. With a data sharing rate of only 4.5% among all studies and 24% among clinical trials, there is clear room for improvement despite the journal's internationally leading stance on encouraging data sharing. Tighter data sharing policies and better incentives for researchers to share their data might be needed.
