# Supplementary material for: Has open data arrived at the British Medical Journal (BMJ)? An observational study
Source: BMJ Open. 2016 Oct 13;6(10):e011784. doi: 10.1136/bmjopen-2016-011784 (PMC5073489; doi:10.1136/bmjopen-2016-011784)
Supplement: supplementary web appendix [file bmjopen-2016-011784supp_appendix.pdf]

Subject Title:
Title of the BMJ research article that was randomly selected (changes per email)

Dear xxxx

My name is Anisa Rowhani-Farid and I am a higher degree research (Masters) student at the School of Public Health and Social Work, Faculty of Health, Queensland University of Technology (QUT) in Brisbane, Australia. The title of my research is: **Promoting a culture of open science and data sharing in health and medical research.**

I am examining how data sharing rates have changed over time and in reaction to data sharing policy changes at the British Medical Journal (BMJ). The BMJ has made several changes to its data sharing policy since 2009.

Your research paper xxxx has been randomly sampled for this study. You mentioned in your data sharing statement that your dataset is available upon request. Would it be possible to receive a copy of your dataset or to be directed to a depository where it is stored? We are interested in re-examining your research dataset in order to quantify the integrity of the data sharing process. We will examine the data to verify that it appears consistent with the published paper (e.g., by checking the sample size), but will not perform a formal re-analysis.

You will not directly benefit from participation in this project however it is hoped the outcomes of the study contribute to the movement toward data sharing in health and medical research. There are minimal risks associated with your participation in this project. These include discomfort associated with your duty to provide data to other researchers. All data and correspondence will be treated with strict confidence and no individual researchers or projects will be mentioned in the paper, rather we will report aggregated statistics. The data will be deleted as soon as we have re-examined the datasets and research data that is generated through this study will be publicly shared in an anonymised format for possible future use.

Please note that this study has been approved by the QUT Human Research Ethics Committee (approval number 1500000842).

QUT is committed to research integrity and the ethical conduct of research projects. However, if you do have any concerns or complaints about the ethical conduct of the project you may contact the QUT Research Ethics Advisory Team on +61 7 3138 5123 or email ethicscontact@qut.edu.au. The QUT Research Ethics Advisory Team is not connected with the research project and can facilitate a resolution to your concern in an impartial manner.

Your response to this email will be taken as consent to participate in this study.

Should you have any further questions about this study, feel free to contact me.

Kind regards

Anisa Rowhani-Farid
Masters Student
anisa.rowhanifarid@hdr.qut.edu.au

Associate Professor Adrian Barnett
Supervisor
a.barnett@qut.edu.au
+61 7 3138 6010

School of Public Health and Social Work, Faculty of Health
Queensland University of Technology
